# The evaluation of AMSR-E soil moisture data in atmospheric modeling using a suitable time series iteration to derive land surface fluxes over the Tibetan Plateau

**DOI:** 10.1371/journal.pone.0226373

**Published:** 2019-12-16

**Authors:** Weiqiang Ma, Yaoming Ma

**Affiliations:** 1 Key Laboratory of Tibetan Environment Changes and Land Surface Processes, Institute of Tibetan Plateau Research, Chinese Academy of Sciences, Beijing, China; 2 CAS Center for Excellence in Tibetan Plateau Earth Sciences, Chinese Academy of Sciences, Beijing, China; 3 University of Chinese Academy of Sciences, Beijing, China; University of the Chinese Academy of Sciences, CHINA

## Abstract

In this study, the initial soil moisture in an atmospheric model was varied by assimilating AMSR-E (The Advanced Microwave Scanning Radiometer for EOS) products, and the results were compared with the default model scenario and in-situ data based on long-term CAMP/Tibet (Coordinated Enhanced Observing Period (CEOP) Asia-Australia Monsoon Project (CAMP) Tibet) observations. The differences between the obtained results (i.e., the new simulation, default model configuration and in-situ data) showed an apparent inconsistency in the model-simulated land surface heat fluxes. The results showed that the soil moisture was sensitive to the specific model simulation. To evaluate and verify the model stability, a long-term modeling study with AMSR-E soil moisture data assimilation was performed. Based on test simulations, AMSR-E data were assimilated into an atmospheric model for July and August 2007. The results showed that the land surface fluxes agreed well with both the in-situ data and the results of the default model configuration. Assimilating the AMSR-E SM products was important for determining the land surface heat fluxes in the WRF model. All the assimilation work substantially improved the modeling of land surface heat fluxes. Land surface heat fluxes are related to atmospheric interactions. Therefore, land surface heat fluxes are very important land surface parameters during these processes. Therefore, the simulation can be used to retrieve land surface heat fluxes from an atmospheric model. It is important to study the surface heating sources that are related to both the water and energy cycles over the Tibetan Plateau.

## Introduction

The Tibetan Plateau is called the third pole [1] in atmospheric and geographic sciences. The atmospheric circulation patterns change over the Tibetan Plateau due to its terrain and surface heating. Therefore, determining the regional land surface heat exchange has become an urgent and necessary topic of research because solar radiation is related to both the energy and water cycles of the Asian monsoon system over the Tibetan Plateau [1–3]. The most important aspect of this topic is the derivation of accurate land surface heat fluxes, as the boundary layer and lower atmosphere are directly affected by land surface heat fluxes. To date, numerous studies have been performed regarding land surface heat fluxes over the Tibetan Plateau [4–14].

Regional-scale land surface heat fluxes are required to better understand the interactions between the land and atmosphere over the Tibetan Plateau. Several methods are typically used to derive regional land surface heat fluxes, including remote sensing, data assimilation, numerical simulations and field observations [11]. For example, remote sensing requires information on the difference between the air temperature and ground surface temperature [15, 16]. However, this method is typically only used in homogeneous areas with moist or semiarid conditions, and there have been very few applications in inhomogeneous high-altitude regions. Therefore, numerical models can be used to simulate regional land surface heat fluxes over heterogeneous land surfaces. Numerical models can be provided with land surface heat fluxes at different resolutions according to the model resolution using many options that are embedded in the weather research and forecasting (WRF) model [17]. The land surface heat fluxes, such as the sensible heat flux and latent heat flux, can then be directly derived from the numerical model output; i.e., regional heat fluxes can be directly simulated with the aid of a numerical model.

In this paper, a numerical model, i.e., the WRF model, was used to simulate regional land surface heat fluxes over the Tibetan Plateau. The purpose of this work was to scale point observations of land surface heat fluxes to the regional scale using the WRF model due to its rigorous physical framework. We changed and assimilated the initial soil moisture (SM) from AMSR-E (the Advanced Microwave Scanning Radiometer-EOS (Earth Observation System)) in the WRF model to improve the determination of land surface fluxes.

## Data and method

### 2.1 Data used

The CAMP/Tibet (Coordinated Enhanced Observing Period Asia-Australia Monsoon Project on the Tibetan Plateau) observational data and AMSR-E remote sensing products were used in this study.

#### 2.1.1 In-situ data

**Fig 1:**
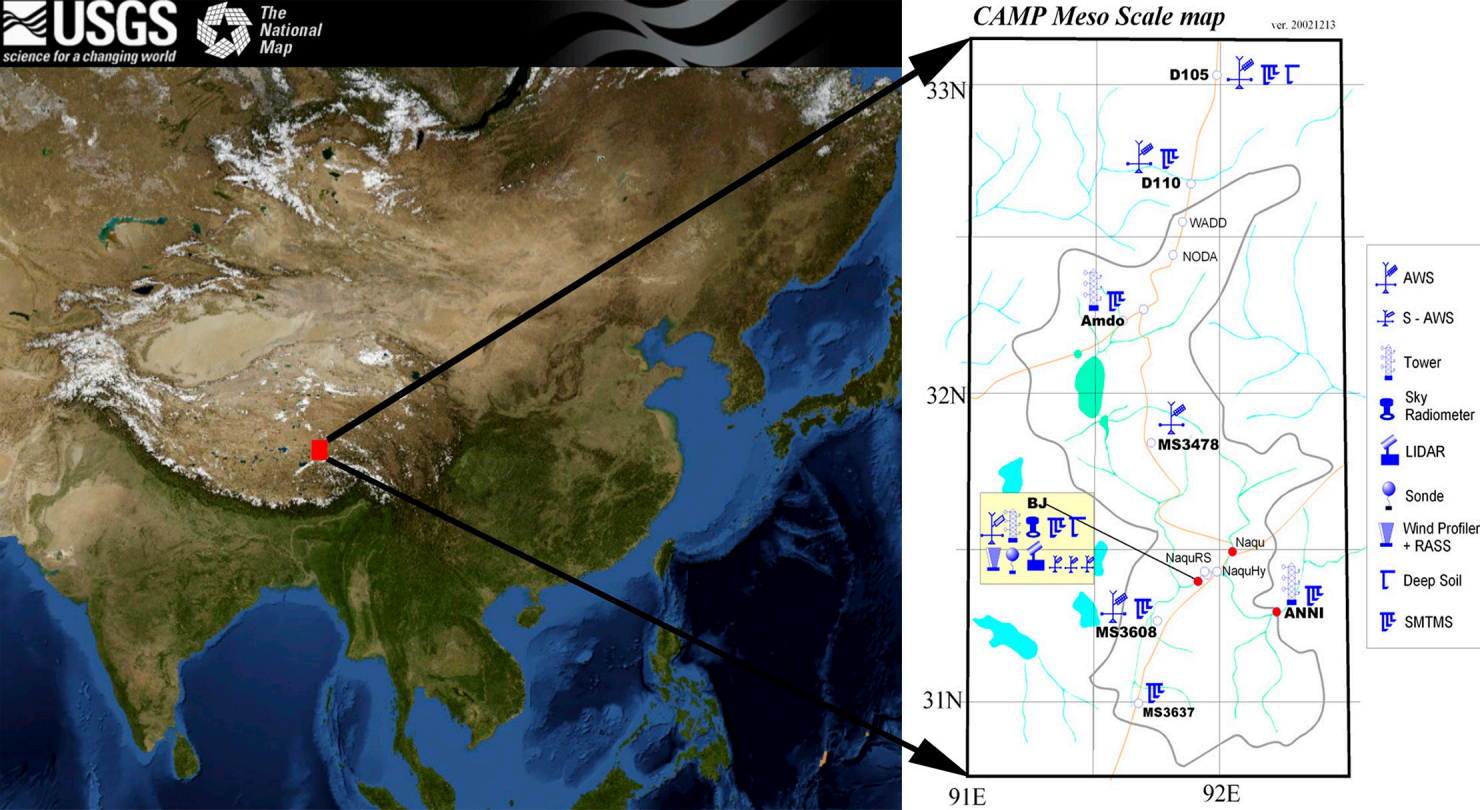
In-situ observation sites within the CAMP/Tibet region. The map was plotted by USGS website. Detailed information concerning the sampling sites is shown in http://viewer.nationalmap.gov/viewer/.

The CAMP/Tibet dataset [11] was used in for the numerical simulation; the in-situ observations were collected at an interval of 60 min. Field observations have been conducted since 1997; accordingly, large amounts of data have been saved and analyzed. The mesoscale observation area was approximately 250×150 km^2^ in this study and consisted of alpine meadows, wetlands, creeks, bare soil and plateau lakes (Fig 1).

The simulation in this study used data from the BJ site (named after the village of Bujiao) to validate the modeling results, including land surface heat fluxes (such as sensible and latent heat fluxes), four components of ground radiation, wind speed and direction, air temperature, SM and temperature in different layers and the land surface status. BJ station, upward and downward shortwave radiation, upward and downward longwave radiation and net radiation are CNR1 Radiometer. For detailed information can be found in Ma 2006 (Ma et al., 2006). The BJ site is located at 32° 22′N, 91° 54′E, i.e., southwest of Ngaqu city. The site has an altitude of 4509 m and is situated within a relatively flat and broad alpine meadow. The land surface status consists of a wide range of classical Tibetan ground characteristics [11].

#### 2.1.2 AMSR-E soil moisture products

We used the AMSR-E SM data products provided by JAXA (http://www.jaxa.jp/index_e.html). These data were used to initialize the SM in the WRF model. The SM contents estimated from the AMSR-E observations were compared with in-situ data at three locations (i.e., Gaize in China, Balranald Bolto in Australia and Little River, GA, in the USA) that exhibit different extents of vegetation coverage. Although the algorithm has a tendency to overestimate in-situ data under precipitating conditions, the estimated values agree well with the changes in the in-situ data at all sites. The results were verified by the comparison of estimated and measured data for three locations with differing vegetation coverage conditions. Compared with results estimated by the Japan Aerospace Exploration Agency standard product version (created by the algorithm before the current revision), the results estimated by the revised algorithm showed a significant improvement in accuracy and reduction in the number of erroneous estimations. In this study, mainly sunny days were selected to ensure that the AMSR-E SM dataset could be trusted [18].

### 2.2 Calculation of land surface fluxes

The model results must be validated using in-situ data. Therefore, we used the combinatory method [19] to calculate the land surface heat fluxes at the BJ site using the following equations. Combinatory method is based on the land surface energy balance. Net radiation is equal to the sum of sensible heat flux, latent heat flux and soil heat flux.

$$H_{0}=\rho C_{p}\kappa^{2}Z_{A}{}^{2}\frac{\partial U}{\partial Z}\frac{\partial \theta }{\partial Z},$$ (1)

$$\lambda E_{0}=\rho \lambda \kappa^{2}Z_{A}{}^{2}\frac{\partial U}{\partial Z}\frac{\partial q}{\partial Z},$$ (2)

$$Z_{A}={\sqrt{Z_{i}Z}}_{i+1},$$ (3)

$$G_{0}=G_{z}+\int_{0}^{z}C_{s}\frac{\partial T}{\partial t}dz,$$ (4)

$$F=\frac{R_{n}-G_{0}}{H_{0}+\lambda E_{0}},$$ (5)

$$H=H_{0}F,$$ (6)

$$\lambda E=\lambda E_{0}F,$$ (7)

where *H*_0_ and *λE*_0_ are the unconverted sensible heat flux and latent heat flux, respectively, *λ* is the latent heat of vaporization, *Z*_*A*_ is the geometric average of *Z*_*i*_ and *Z*_*i+1*_, *C*_*s*_ is the soil volumetric heat capacity, *G*_*z*_ is the surface-observed soil heat flux observed below z (z = 10 cm in this study), *κ* is the *Karman* constant, *θ* is the megadyne temperature and *q* is the specific humidity. Using Eqs (4), (6) and (7), the land surface heat fluxes can be directly calculated.

### 2.3 Error analysis tools

The absolute percentage difference (APD) can be used to quantitatively measure the difference between model-derived results (*H*_*derived(i)*_) and in-situ observations (*H*_*measured(i)*_) using the following equation:

$$APD=\frac{\left|H_{derived\;(i)}-H_{measured\;(i)}\right|}{H_{measured\;(i)}}.$$ (8)

### 2.4 Model and sensitivity experiments

**Fig 2:**
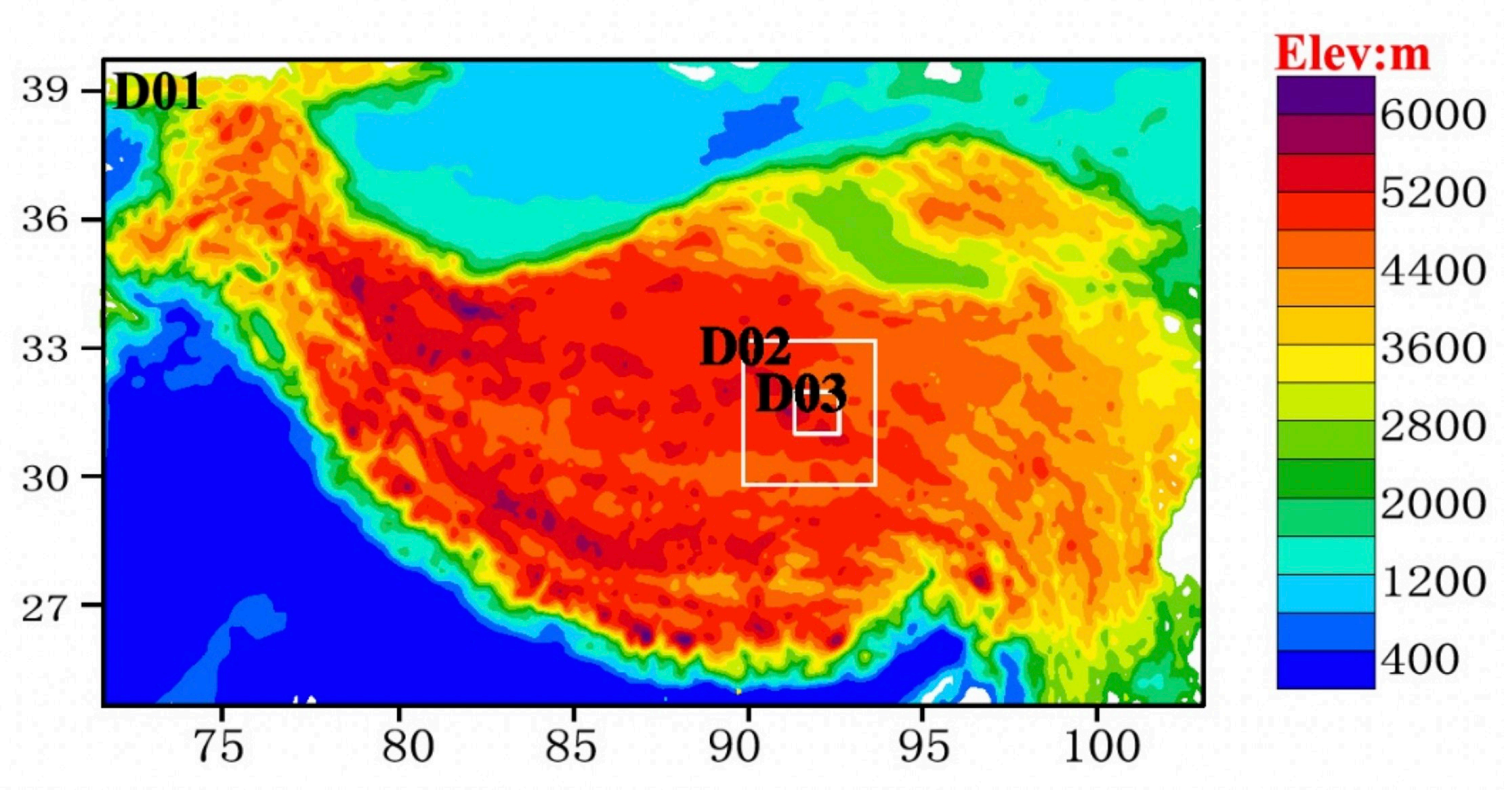
Nested domains used in the WRF model simulations.

The WRF modeling system was used in this study. The WRF model is in the public domain and is freely available for community use. The model is designed to be a flexible state-of-the-art atmospheric simulation system that is portable and efficient on available parallel computing platforms. Moreover, the WRF model is suitable for use in a broad range of applications that span scales ranging from meters to thousands of kilometers. The WRF model has been developed and maintained by the National Center for Atmospheric Research (NCAR), the National Oceanic and Atmospheric Administration (the National Centers for Environmental Prediction (NCEP) and the Forecast Systems Laboratory (FSL)), the Air Force Weather Agency (AFWA), the Naval Research Laboratory, the University of Oklahoma, and the Federal Aviation Administration (FAA) (see http://www.mmm.ucar.edu/wrf/users/) [17]. A triple-nested grid system centered at 31.37° N, 91.89° E was used in this study (Fig 2). The BJ site is located in the center of domain 3.

Based on a series of tests, a set of physical options were selected for subsequent WRF simulations. The simulations used the Kessler microphysics scheme. The Rapid Radiative Transfer Model (RRTM) and the Dudhia shortwave radiation scheme were used to calculate the longwave and shortwave radiation, respectively, as well as their transfer within the atmosphere. The surface layer physics was based on the Monin-Obukhov and standard similarity functions. Moreover, the Noah land surface model was chosen for the land surface physics model, and the Yonsei University scheme was selected for the planetary boundary layer physics. Cumulus clouds were simulated using the Kain-Fritsch convection scheme [20–22] in domains 1 and 2; no convection scheme was applied in domain 3.

The simulations began at 0000 UTC 1 July 2007 and ended at 0000 UTC 30 August 2007 (encompassing 61 days). The 6-h NCEP FNL analysis dataset from the Global Forecast System (GFS) provided both the initial and lateral boundary conditions for the WRF simulations. The resolution of FNL data is 0.25 degree. We used WPS in WRF model to process the same resolution to run the model. The configurations of the nested domains are listed in Table 1.

**Table 1:** Nested domain for the modeling area.

Nest	Domain 3 center	Grid points	Resolution (km)	Time step (s)	Grid spacing resolution
1	91°54′E 31°22′N	354×193	9	60	10 m
2		139×127	3	20	5 m
3		124×112	1	4	30 s

**Case 1:** This experiment used the default values for all parameters of the WRF model. The domain was designed based on the USGS (United States Geological Survey) 1-km terrain data, and the soil parameters were obtained from the National Centers for Environmental Prediction (NECP) FNL (DOI: 10.5065/D6M043C6) analysis dataset (Wei Wang et al., 2012).

**Case 2:** In this case, the initial SM values were changed in the first layer of WRF model using the Noah land surface model and based on AMSR-E SM data. After checking the initial SM values of the model, we found that the model SM values were higher than the observed values. Therefore, the SM of the WRF model was transformed using AMSR-E SM data in this case. Because the Nagqu area is relatively flat and consists of a uniform land surface, the AMSR-E SM data can be used to represent a large area of the Tibetan Plateau. Therefore, we replaced the SM data in the first layer with the Noah SM data; for example, SM was replaced with SM000010 in the WRF model. That is to say AMSR-E products can deirctly replace the FNL dataset in the model WPS part every 6 hours. The objective of case 2 was to identify the effects of SM initialization on the modeling results and to demonstrate that the model results are similar to the actual land heat fluxes of the northern Tibetan Plateau.

## Results and discussion

The assimilation experiment described above was applied to the Tibetan Plateau BJ site. The modeling results were compared with the in-situ data. The analysis period was from July 1 2007 to August 30 2007.

### 3.1 Differences in the modeling results

**Fig 3:**
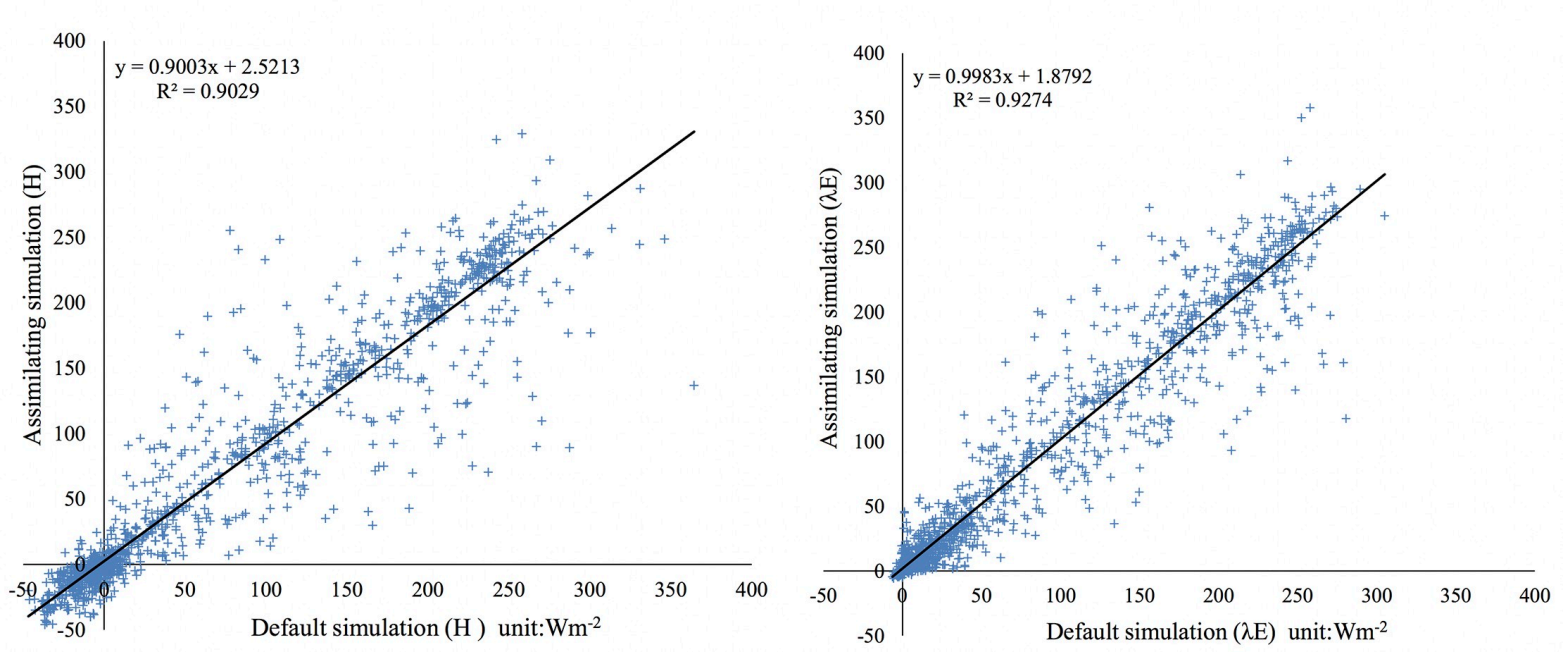
Scatter diagrams of the sensible heat flux H and latent heat flux λE estimated using the default and assimilation WRF model simulations (the 1:1 line is shown as a black line).

To examine the differences between the default and assimilation simulations, scatter diagrams of the monthly sensible and latent heat fluxes estimated from the default simulation (x-axis) versus the assimilation simulation (y-axis) are presented. The simulation period was from 1 July 2007 to 30 August 2007. The 1:1 line is shown in the Fig 3.

Fig 3 demonstrates that the assimilation experiment exhibited large differences relative to the default experiment. The modeled sensible and latent heat fluxes changed when 6-h AMSR-E SM data were assimilated in the WRF model, showing an improvement of the modeled land surface heat fluxes using the data assimilation. An examination of the default SM values of the WRF model showed that there were large differences between the model and AMSR-E data. The AMSR-E data were validated using in-situ data over the Tibetan Plateau. Therefore, we believe that the assimilation experiment provides a large improvement over pre-existing land heat flux information. Additional details are provided in section 3.2.

### 3.2 Daily sensible and latent heat fluxes

**Fig 4:**
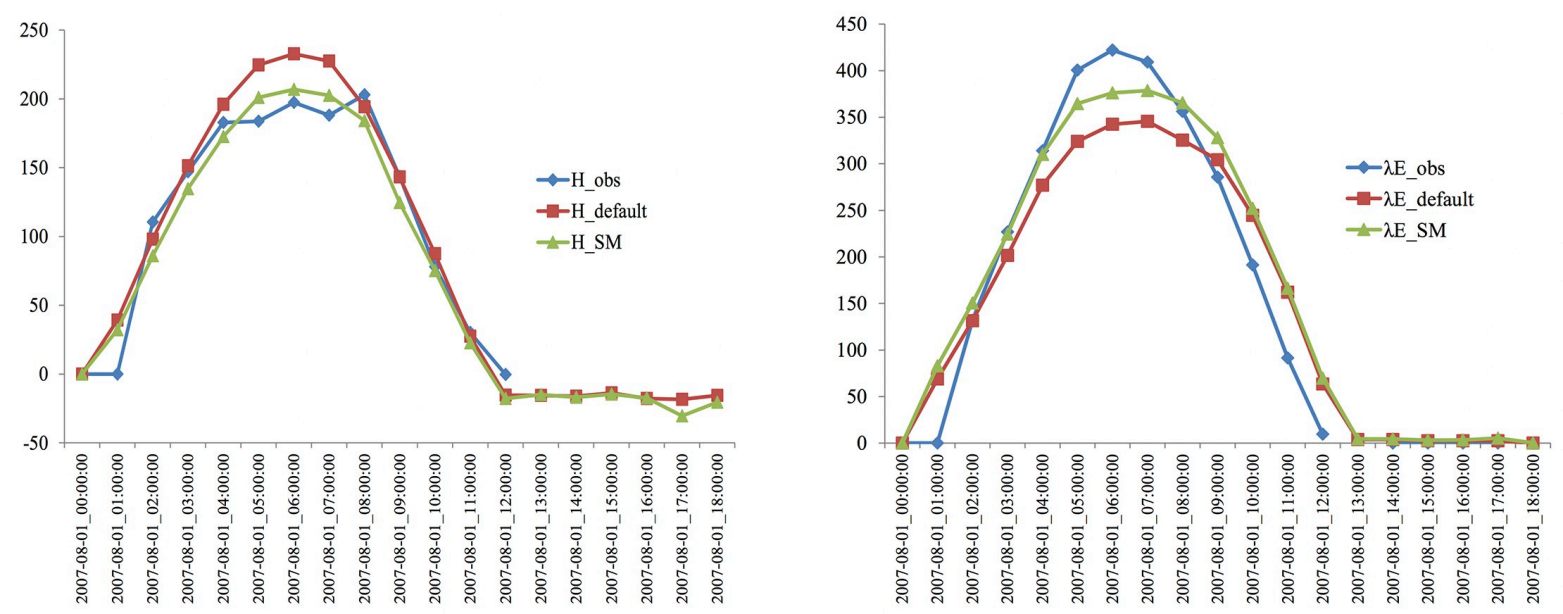
Comparisons of the sensible and latent heat fluxes obtained from the in situ data, default model configuration and assimilation experiment (units: Wm-2).

To distinguish differences among the in-situ data for the default model configuration and the assimilation experiment, the daily sensible and latent heat fluxes were compared. Plots of the daily variations in the observed and modeled sensible and latent heat fluxes are shown in Fig 4.

**Fig 5:**
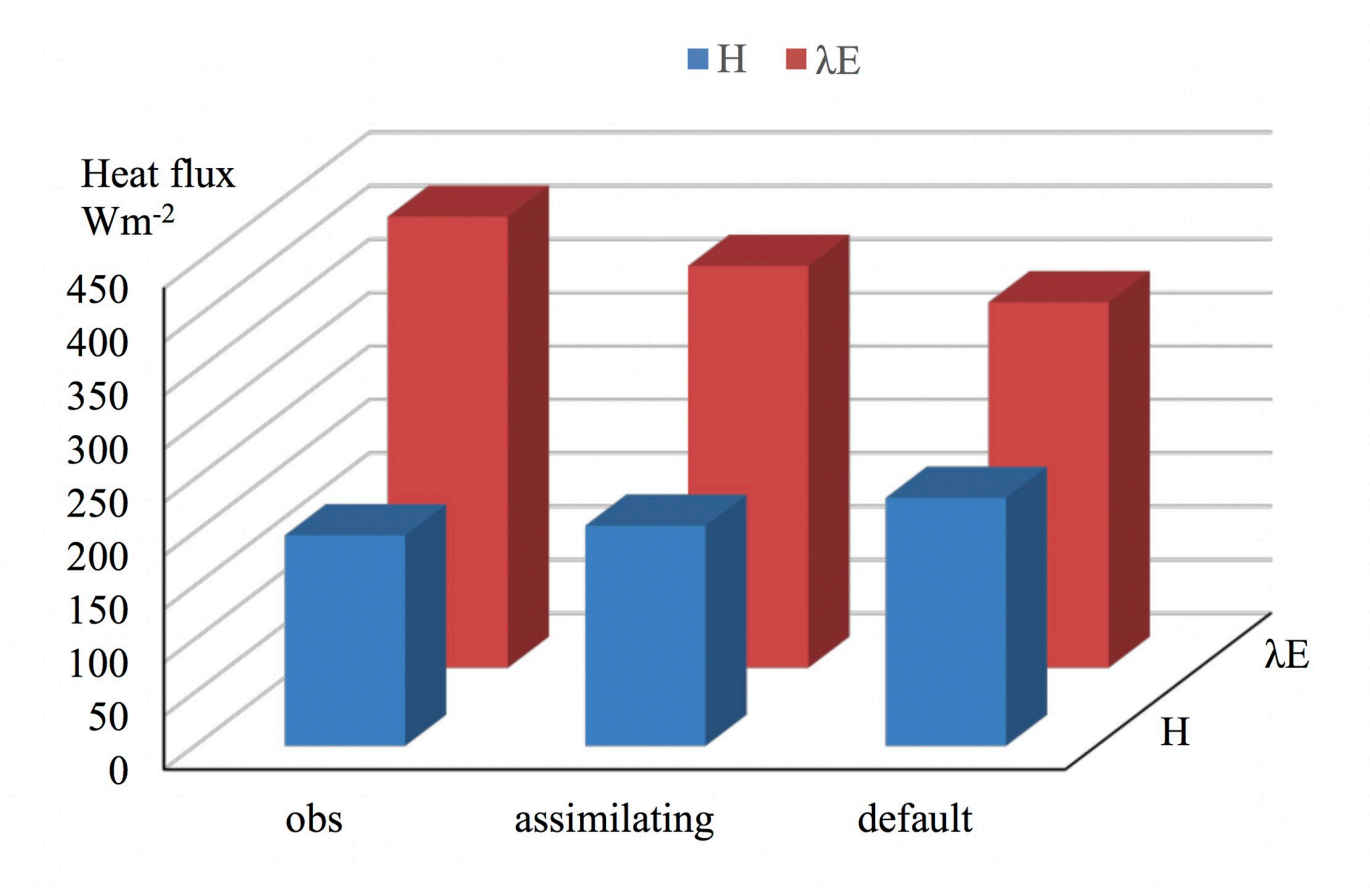
Comparisons of the mean sensible and latent heat fluxes obtained from the in situ data, default model configuration and assimilation experiment.

The sensible and latent heat fluxes over the Tibetan Plateau predicted using the model with assimilation showed better agreement with the field measurements compared to the results of the default model (Fig 5). Based on the assimilation experiment results and observations, the mean APDs were 4.5% and 10% for the sensible and latent heat fluxes, respectively; however, for the default experiment, the APDs were 17.7% and 18.9% for the sensible and latent heat fluxes, respectively. Therefore, the assimilation technique for retrieving land surface heat fluxes is reasonable and can used for the Tibetan Plateau. The fluxes derived from the assimilation experiment are within a reasonable range.

### 3.3 Land surface fluxes using long time series

**Fig 6:**
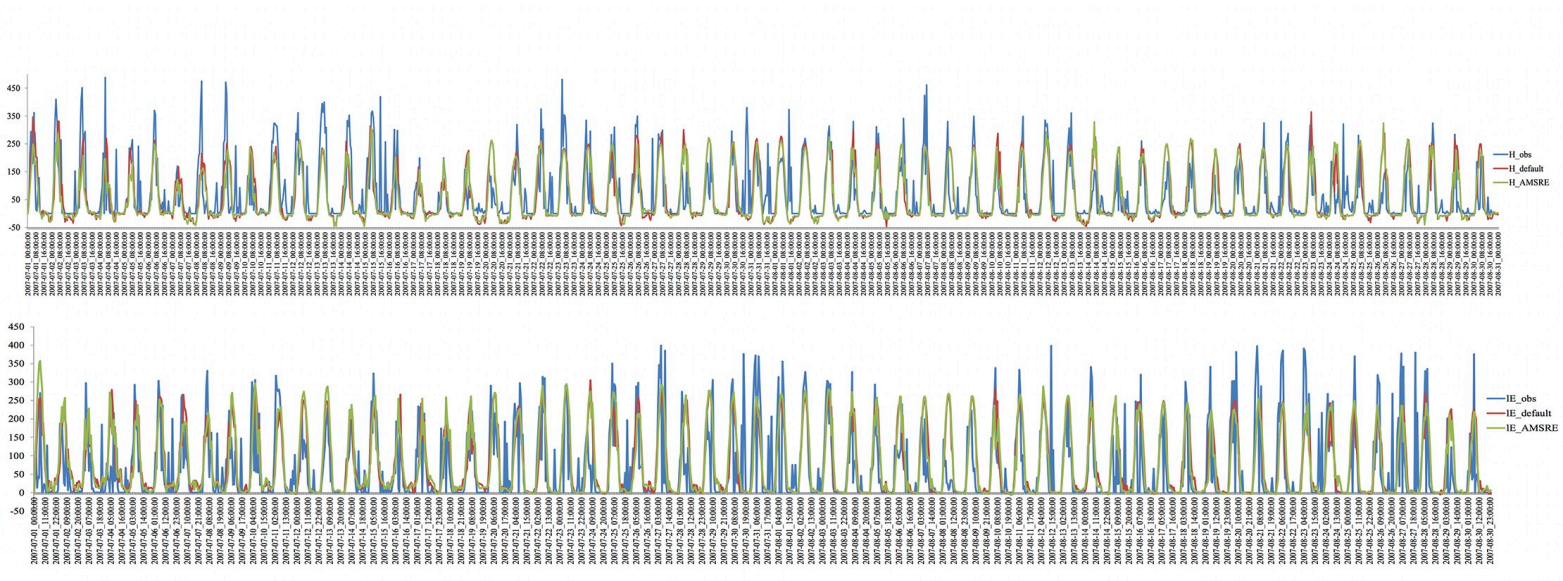
Comparisons of the sensible and latent heat fluxes obtained from the in situ data, default model configuration results and assimilation experiment (units: Wm-2).

To evaluate the stability of the WRF model, a long time series experiment spanning from July 1 to August 30 was performed. The combinatory method [19] was applied to calculate the sensible and latent heat fluxes using ground-truth in-situ observational data. The long-term series for the land surface heat fluxes are shown in Fig 6. The diurnal variations in the land surface heat fluxes were accurately simulated; i.e., the modeled and observed data showed identical trends.

The AMSR-E SM products were assimilated into the WRF model using initial boundary conditions. Fig 6 demonstrates that the 2-month simulated sensible and latent heat fluxes compare well with the CAMP/Tibet observations. Moreover, the detailed daily comparison presented in section 3.2 showed that the assimilation experiment results were highly similar to the in-situ observations. However, the application of the assimilation experiment to simulate the long-term evolution of land surface heat fluxes was not a simple task even when using AMSR-E SM products. The long-term simulations required the use of validated AMSR-E SM products and reasonable inputs for the WRF model. After examining the initial SM in the WRF model, we found that the SM in the model was substantially larger than the observations. Therefore, assimilating the AMSR-E SM products was important for determining the land surface heat fluxes in the WRF model. All of the assimilation work substantially improved the modeling of land surface heat fluxes.

## Conclusions

We assimilated AMSR-E SM products into the WRF model over the Tibetan Plateau. These SM products were validated using observations. Hence, these products can be safely used in WRF model simulations. In the present study, we tested and assimilated AMSR-E SM products, which can be used to reduce the modeled heat flux bias in long-term simulations.

After analyzing the simulation results, especially for the assimilation test, we found that the WRF model successfully simulated the land surface heat fluxes over the Tibetan Plateau with a diurnal variation. The modeling results agreed well with the observations. Based on the observations, when the initial model values are assimilated using the AMSR-E SM products, the WRF model can reproduce changes in heat fluxes.

When the initial SM field was replaced with the AMSR-E SM data, the model-simulated sensible and latent heat fluxes were improved; specifically, the simulation results agreed well with the observed “true values”.

However, even with the adoption of the WRF model for simulating land surface heat fluxes over the Tibetan Plateau, some errors remain. It means that some discrepancies still exist between model simulation results and in situ measurements. Future studies will focus on the initial field and the addition of more parameters in the simulations to obtain high-quality land surface fluxes. Land surface heat fluxes are related to atmospheric interactions. Therefore, land surface heat fluxes are very important land surface parameters during these processes. Therefore, it is important to study the surface heating sources that are related to both the water and energy cycles over the Tibetan Plateau.
